# Timing and Modality of Kidney Replacement Therapy in Children and Adolescents

**DOI:** 10.1016/j.ekir.2024.06.009

**Published:** 2024-06-12

**Authors:** Julia Thumfart, Steffen Wagner, Marietta Kirchner, Karolis Azukaitis, Aysun K. Bayazit, Lukasz Obrycki, Nur Canpolat, Ipek Kaplan Bulut, Ali Duzova, Ali Anarat, Lucie Bessenay, Rukshana Shroff, Dusan Paripovic, Lale Sever, Cengiz Candan, Francesca Lugani, Alev Yilmaz, Fatos Yalcinkaya, Klaus Arbeiter, Aysel Kiyak, Aleksandra Zurowska, Matthias Galiano, Uwe Querfeld, Anette Melk, Franz Schaefer, 4C Study Group, G. Cortina, K. Arbeiter, J. Dusek, J. Harambat, B. Ranchin, M. Fischbach, U. Querfeld, S. Habbig, M. Galiano, R. Büscher, C. Gimpel, M. Kemper, A. Melk, D. Thurn, F. Schaefer, A. Doyon, E. Wühl, M. Pohl, S. Wygoda, N. Jeck, B. Kranz, M. Wigger, G. Montini, F. Lugani, S. Testa, E. Vidal, C. Matteucci, S. Picca, A. Jankauskiene, K. Azukaitis, A. Zurowska, D. Drodz, M. Tkaczyk, T. Urasinski, M. Litwin, M. Szczepanska, A. Texeira, A. Peco-Antic, B. Bucher, G. Laube, A. Anarat, A.K. Bayazit, F. Yalcinkaya, E. Basin, N. Cakar, O. Soylemezoglu, A. Duzova, Y. Bilginer, H. Erdogan, O. Donmez, A. Balat, A. Kiyak, S. Caliskan, N. Canpolat, C. Candan, M. Civilibal, S. Emre, G. Ozcelik, S. Mir, B. Sözeri, O. Yavascan, Y. Tabel, P. Ertan, E. Yilmaz, R. Shroff

**Affiliations:** 1 Department of Pediatric Gastroenterology, Nephrology and Metabolic Diseases, Charité Universitätsmedizin, Berlin, Germany; 2 BHT Berliner Hochschule für Technik, Berlin, Germany; 3 Institute of Medical Biometry, University of Heidelberg, Heidelberg, Germany; 4 Clinic of Pediatrics, Institute of Clinical Medicine, Vilnius University, Vilnius, Lithuania; 5 Department of Pediatric Nephrology, School of Medicine, Cukurova University, Adana, Türkiye; 6 Department of Nephrology, Kidney Transplantation and Hypertension, Children`s Memorial Health Institute, Warsaw, Poland; 7 Division of Pediatric Nephrology, Istanbul University Cerrahpasa Faculty of Medicine, Istanbul, Türkiye; 8 Department of Pediatric Nephrology, Ege University Faculty of Medicine, Izmir, Türkiye; 9 Division of Pediatric Nephrology, Department of Pediatrics, Hacettepe University Faculty of Medicine, Ankara, Türkiye; 10 Department of Pediatric Nephrology, School of Medicine, Cukurova University, Adana, Türkiye; 11 Hôpital Femme Mère Enfant, Hospices Civils de Lyon, Université de Lyon, Lyon, France; 12 UCL Great Ormond Street Hospital and Institute of Child Health, London, UK; 13 Department of Pediatric Nephrology, University Children's Hospital, Belgrade, Serbia; 14 Department of Pediatric Nephrology, Istanbul Medeniyet University, Istanbul, Türkiye; 15 Division of Nephrology and Transplantation, IRCCS Istituto G. Gaslini, Genova, Italy; 16 Istanbul University, Istanbul Faculty of Medicine, Istanbul, Türkiye; 17 Department of Pediatrics, Ankara University Medical School, Ankara, Türkiye; 18 Division of Pediatric Nephrology and Gastroenterology, Department of Pediatrics and Adolescent Medicine, Medical University of Vienna, Vienna, Austria; 19 Department of Pediatric Nephrology, Yenimahalle Egitim ve Arastirma Hastanesi Bakirkoy, Istanbul, Türkiye; 20 Department of Pediatric Nephrology, Medical University of Gdansk, Gdansk, Poland; 21 Department of Pediatrics and Adolescent Medicine, University of Erlangen-Nürnberg, Erlangen, Germany; 22 Department of Pediatric Kidney, Liver and Metabolic Diseases, Hannover Medical School, Hannover, Germany; 23 Center for Pediatrics and Adolescent Medicine, University Hospital Heidelberg, Heidelberg, Germany

**Keywords:** dialysis initiation, pediatric risk factors, progression kidney disease

## Abstract

**Introduction:**

The choice and timing of kidney replacement therapy (KRT) is influenced by clinical factors, laboratory features, feasibility issues, family preferences, and clinicians' attitudes. We analyzed the factors associated with KRT modality and timing in a multicenter, multinational prospective pediatric cohort study.

**Methods:**

A total of 695 pediatric patients with chronic kidney disease (CKD) enrolled into the Cardiovascular Comorbidity in Children with CKD (4C) study at age 6 to 17 years with estimated glomerular filtration rate (eGFR) of 10 to 60 ml/min per 1.73 m^2^ were investigated. Competing risk regression was performed to identify factors associated with initiation of dialysis or preemptive transplantation (Tx), including primary renal diagnosis, demographics, anthropometrics, and laboratory parameters.

**Results:**

During the 8-year observation period, 342 patients (49%) started KRT. Of these, 200 patients started dialysis, whereas 142 patients underwent preemptive Tx. A lower eGFR at enrolment (Hazard ratio [HR]: 0.76 [95% confidence interval: 0.74–0.78]), a steeper eGFR slope (HR: 0.90 [0.85–0.95], and a higher systolic blood pressure SD score (SDS) (HR: 2.07 [1.49–2.87]) increased the likelihood of KRT initiation. Patients with glomerulopathies were more likely to start dialysis than children with congenital anomalies of the kidneys and urinary tracts (CAKUT) (HR: 3.81 [2.52–5.76]). Lower body mass index (BMI) SDS (HR: 0.73 [0.6–0.89]) and lower hemoglobin (HR: 0.8 [0.72–0.9]) were associated with higher likelihood of dialysis. A significant center effect was observed, accounting for 6.8% (dialysis) to 8.7% (preemptive Tx) of explained variation.

**Conclusion:**

The timing and choice of KRT in pediatric patients is influenced by the rate of kidney function loss, the underlying kidney disease, nutritional status, blood pressure, anemia and center-specific factors.

## Graphical abstract

**Figure:**
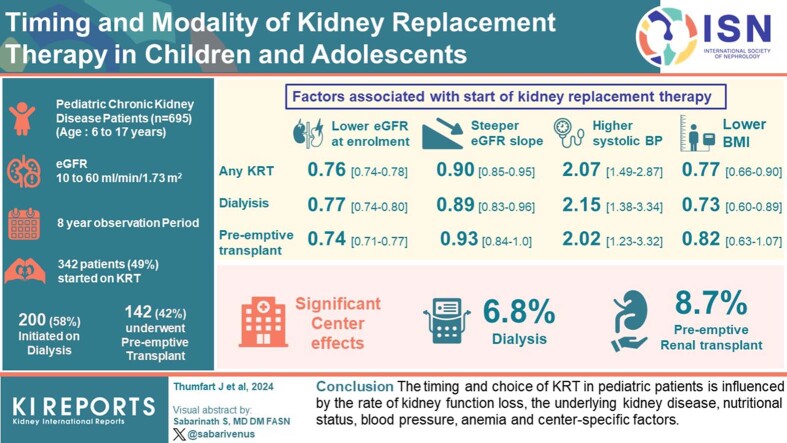

The optimal timing of the start of KRT in patients with kidney failure is controversial. Dialysis improves some but not all complications of CKD and causes treatment-related complications.^1^ The KRT option more frequently available in the pediatric CKD population is preemptive Tx. It is associated with better patient survival^2^ and reduced cardiovascular comorbidity,^3^ but advances the need for maintenance immunosuppressive therapy and also causes various long-term sequelae. In an analysis of pediatric patients followed-up in the US Renal Data System, a higher GFR at dialysis initiation was associated with a lower survival rate, particularly among patients whose initial dialysis modality was hemodialysis.^4^^,^^5^ The ERA-EDTA/ESPN registry study found no difference in mortality rates between children with early versus late start of dialysis,^6^ in keeping with randomized^7^ and observational^8^, ^9^, ^10^ trial evidence in adults. In children followed-up in the Australia and New Zealand Dialysis and Transplant Registry, a trend toward earlier KRT initiation was observed over several decades, which was not accompanied with changes in patient survival.^11^

International guidelines^12^ and recommendations^1^ propose to base the decision to initiate KRT primarily on clinical signs and symptoms such as hypertension, malnutrition, growth retardation, and impaired physical performance rather than a specific level of kidney function.

Here, we analyzed a large prospective European pediatric CKD cohort (the 4C Study^13^) to explore the main factors associated with the decision to initiate KRT in general, and dialysis versus preemptive Tx in particular, in current clinical practice.

## Methods

### Study Population and Protocol

For this analysis, 695 patients with at least 2 visits before initiation of KRT were selected from the 4C study cohort. The 4C study is a prospective observational study that enrolled pediatric patients with CKD aged between 6 and 17 years with an eGFR of 10 to 60 ml/min per 1.73 m^2^ not yet receiving KRT at 55 study sites in 12 European countries and Türkiye between 2009 and 2012.^13^ The patients were followed-up with by 6-monthly study visits until May 2018. The primary renal diagnoses were categorized as CAKUT, glomerulopathies, tubulointerstitial diseases, postacute kidney injury CKD, and others/unknown. Every 6 months, the medical and medication history, anthropometric, and blood pressure as well as blood and urine samples were obtained. The study was designed and performed according to the Declaration of Helsinki. Protocols were approved by the central ethics committee of Heidelberg University Medical Faculty and by each local ethical committee. Written informed consent was obtained from the parents and adolescents.

### Laboratory Analysis

Biochemical parameters, including serum creatinine, phosphate, and calcium were performed centrally using standard laboratory techniques. Serum potassium, bicarbonate, intact parathyroid hormone, and hemoglobin measurements were determined locally. eGFR was estimated using the bedside Schwartz formula.^14^

### Statistical Analysis

The last documented visit before starting KRT was investigated. Potentially significant differences between groups (dialysis vs. preemptive Tx) were identified using *t* test or Welch test for normally distributed, and Wilcoxon rank-sum test for nonnormally distributed continuous variables. Chi-square test was used for categorical variables. *P* < 0.05 was considered statistically significant. Data are given as median (interquartile range) or frequencies (*n* and %).

The impact of the various variables on pre-KRT time-to-event time was analyzed using a time-resolved proportional hazard model to investigate any KRT as outcome and a time-resolved competing risk model,^15^ with dialysis and preemptive kidney Tx as competing events, for KRT specific analysis. Variables considered in the modeling process included time-independent variables such as primary renal diagnosis, sex (male and female) and parental ethnic background, and time-dependent variables, including eGFR, eGFR slope, age, BMI SDS, height SDS, systolic blood pressure SDS, number of antihypertensive drugs, hospitalization rate, the presence of comorbid conditions (e.g., intellectual impairment), physical activity, hemoglobin, serum potassium, bicarbonate, calcium, phosphate, and intact parathyroid hormone levels. The time-resolved eGFR slope was determined by applying a linear mixed-effects model to the eGFR values observed within the preceding 2 years. Random effects were considered for level of eGFR and its annual change.^16^ Variable selection was performed using an AIC-based stepwise algorithm. The significance of the remaining regressors with respect to model fit was determined via a likelihood ratio test.

All models considered possible nonlinear relationships between variables and hazard function using smoothing splines for the numeric variables. According to the shape and confidence intervals of the spline estimates, the regressors were split into intervals in a second step for the final analysis, so that nonlinear behavior was still considered and direct interpretability in terms of HRs for the so-applied piecewise linear approximations was obtained.

The modeling approach also considered a random effect using a frailty term for investigator site to capture confounding at center level. Center effects were expressed as the fraction of explained variation of the respective survival model (R^2^_D_).^17^ In addition, the SD of the estimated random effect was used to quantify the corresponding HR based on the underlying random effects normality assumption, that is, one expects 15% of the centers to be 1 SD or more above and 15% of the centers to be 1 SD or more below the mean, leading to HR exp (+/− 1 SD).

Missing values were imputed using 10 multiple imputations by chained equations.^18^ Because of the longitudinal character of the data, a random effect for patients was considered during imputation. Imputed values were postprocessed so that only values within a medically meaningful range were accepted. Data were complete for age, primary renal diagnosis, number of antihypertensive drugs, sex, height and center. Blood pressure, ethnicity, and BMI were available in >99% of observations. Missing of laboratory data ranged from 7.6% to 15.3%. All statistical analyses were performed using the R statistical software version 4.3.1 (The R Foundation for Statistical Computing, Vienna, Austria).^19^ The R packages survival v3.5-7, mice v3.16.0, and lme4 1.1-34 were used.^15^^,^^18^^,^^20^

## Results

### Characteristics of Study Cohort

The characteristics of the study cohort are given in Table 1. During the study period, 342 patients (49%) started KRT. Of these, 200 patients (58%) commenced dialysis (107 hemodialysis and 93 peritoneal dialysis) and 142 patients (42%) underwent preemptive Tx (101 living-related and 41 deceased-donor donations). Among the 200 patients who started dialysis, 12 patients received a kidney transplant (7 living-related and 5 deceased-donor allografts within 3 months after starting dialysis).

**Table 1:** Patient characteristics and group differences before starting kidney replacement therapy

Characteristics	All patients (*N* = 695)	Any KRT (*n* = 342)	Dialysis (*n* = 200)	Preemptive Tx (*n* = 142)
Age (yr)	12.3 (5.6)	14.4 (4.1)	14.5 (4.3)	14.2 (3.5)
Female sex	243 (35%)	122 (35.7%)	78 (39%)	44 (31%)
Primary renal diagnosis				
CAKUT	477 (68.6%)	212 (61.8%)	119 (59.5%)	93 (65.5%)
Glomerulopathy	59 (8.5%)	35 (10.2%)	28 (14%)	7 (4.9%)
Post-AKI CKD	34 (4.9%)	17 (5%)	9 (4.5%)	8 (5.6%)
Tubulointerstitial disease	94 (13.5%)	61 (17.8%)	33 (16.5%)	28 (19.7%)
Other/unknown	31 (4.5%)	17 (5%)	11 (5.5%)	6 (4.2%)
Comorbidities				
0	321 (46.2%)	138 (40.4%)	75 (37.5%)	63 (44.4%)
≥1	374 (53.8%)	204 (59.5%)	125 (62.5%)	76 (53.5%)
Physical activity				
none	157 (23.1%)	114 (33.2%)	68 (34%)	46 (32.4%)
1–4 h/wk	241 (35.5%)	110 (32.1%)	60 (30%)	50 (35.2%)
>4 h/wk	281 (41.4%)	104 (30.4%)	63 (31.5%)	41 (28.9%)
BMI SDS	0.19 (1.55)	0.19 (1.56)	0.19 (1.6)	0.18 (1.49)
Height SDS	−1.24 (1.63)	−1.22 (1.85)	−1.44 (2.14)	−0.96 (1.63)
Δ height SDS/yr	—	−0.043 (0.51)	−0.051 (0.6)	−0.038 (0.41)
Systolic blood pressure SDS	0.71 (1.63)	0.95 (1.63)	0.95 (1.64)	0.94 (1.68)
Δ systolic blood pressure SDS/yr	—	0.324 (3.19)	0.107 (3.13)	0.56 (3.01)
Diastolic blood pressure SDS	0.58 (1.27)	0.69 (1.46)	0.77 (1.61)	0.66 (1.27)
Δ diastolic blood pressure SDS/yr	—	0.004 (2.45)	0.113 (2.29)	−0.02
No. of antihypertensive drugs	1 (1)	1 (2)	1 (2)	1 (1)
eGFR (ml/min per 1.73 m^2^)	25.6 (16.8)	11.6 (5.1)	11.8 (5.2)	11.1 (5.1)
Δ eGFR (ml/min per 1.73m^2^/yr)	—	−5.0 (8.6)	−5.5 (8.9)	−4.8 (7.7)
Hemoglobin (g/dl)	11.7 (2.2)	10.9 (1.8)	10.8 (1.9)	11.1 (1.9)
Serum urea (g/dl)	43.9 (26.5)	74 (49.3)	76.1 (47.6)	72.8 (53.8)
Serum bicarbonate (mmol/l)	21.3 (4.6)	21.2 (4.8)	21 (5.13)	21.4 (4.6)
Serum calcium (mmol/l)	2.27 (0.21)	2.33 (0.34)	2.29 (0.3)	2.38 (0.35)
Serum phosphorus (mmol/l)	1.5 (0.36)	1.73 (0.5)	1.78 (0.54)	1.7 (0.42)
Serum iPTH (pmol/l)	13.5 (16.5)	24.4 (36.5)	26.8 (36)	18.7 (27.2)
Serum potassium (mmol/l)	4.5 (0.8)	4.5 (1)	4.5 (0.9)	4.5 (0.9)

CAKUT, congenital anomalies of the kidney and urinary tract; eGFR, estimated glomerular filtration rate; iPTH, intact parathyroid hormone; KRT, kidney replacement therapy; post-AKI CKD, post-acute kidney injury chronic kidney disease; SDS, SD score; Tx, kidney transplant.

Data were obtained at study entry (first column) and at the last visit before start of KRT (columns 2–4).

Data represent absolute numbers (%), median (interquartile range).

Δ annualized change in variables calculated from 2 last measurements before KRT.

**Figure 1:**
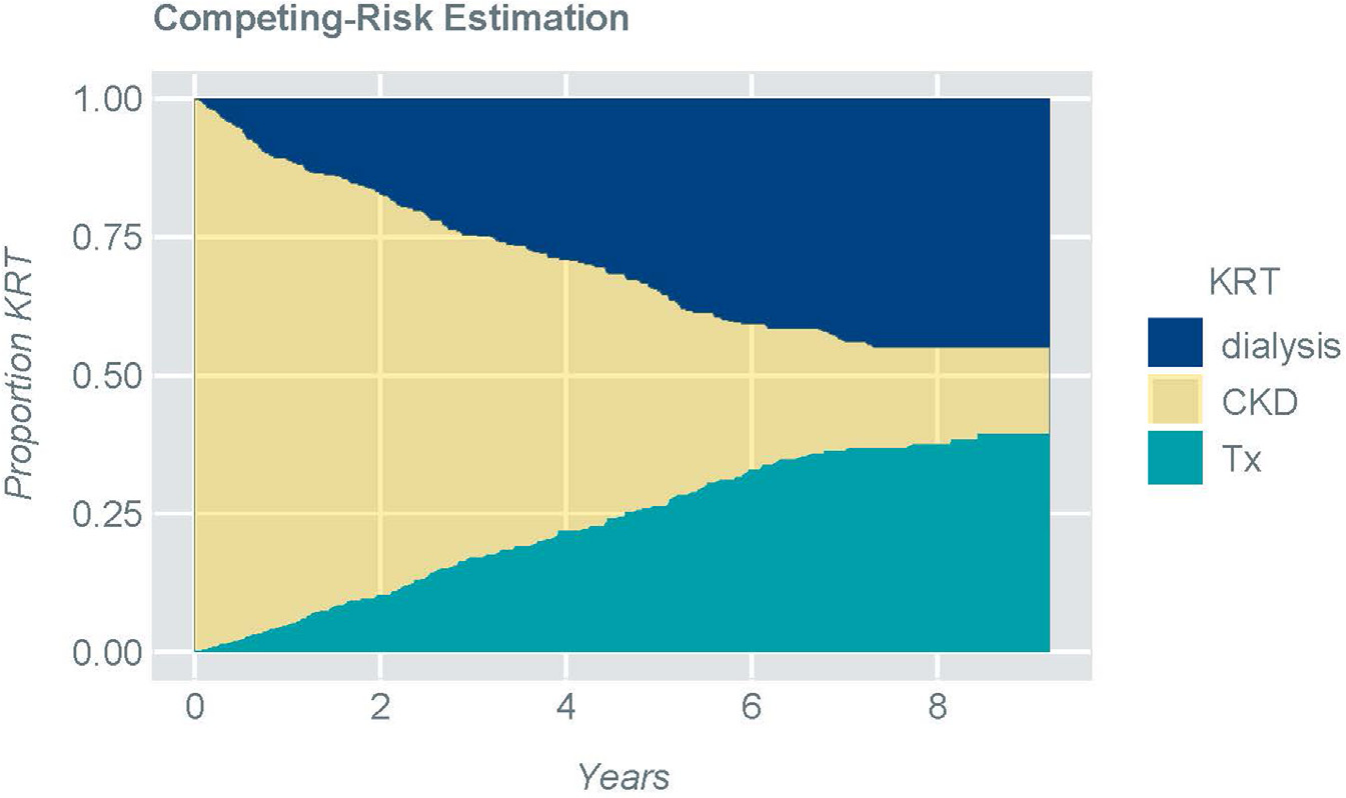
Competing risk analysis addressing the cumulative likelihood of starting dialysis, receiving a preemptive transplant or remaining on conservative therapy (CKD) CKD, chronic kidney disease; KRT, kidney replacement therapy; Tx, transplantation.

Censoring events were observed for 353 patients (CKD group): death (*n* = 3; 0.8%), loss to follow-up (*n* = 106; 30%), transition to adult clinic (*n* = 83; 24%), patient's wish (*n* = 37; 10%), other reasons (*n* = 33; 9%) and end of study (*n* = 91; 26%). The progressive likelihood to start dialysis or receive a preemptive Tx during the observation period is shown in Figure 1.

KRT was started at a median age of 14.4 years at a median eGFR of 11.6 ml/min per 1.73 m^2^ (Table 1). The median time interval between the last pre-KRT study visit and start of KRT was 72 days in the dialysis group and 100 days in the Tx group (*P* = 0.004). Taking into account the average rate of eGFR loss and the time interval between last follow-up visit and start of KRT, patients started dialysis with a median eGFR of 11 ml/min per 1.73 m^2^ (interquartile range: 4.9) and received a preemptive Tx with a median eGFR of 10.7 ml/min per 1.73 m^2^ (interquartile range: 4.6).

Relative to the patients who started dialysis, the children who underwent preemptive Tx did not differ from those who commenced dialysis in terms of age (*P* = 0.69), sex distribution (*P* = 0.16), primary renal diagnosis (*P* = 0.09), comorbidities (*P* = 0.17), urea level (*P* = 0.71), eGFR (*P* = 0.25), or the rate of eGFR loss (*P* = 0.27). However, the children who underwent Tx had significantly higher hemoglobin (*p* = 0.008) and serum calcium (*P* = 0.004), and lower intact parathyroid hormone levels (*P* = 0.04) (Table 1). They were also significantly taller (*P* = 0.007) but did not differ in pre-KRT growth velocity (*p* = 0.49). Neither blood pressure (systolic *P* = 0.25, diastolic *P* = 0.14) nor the change in blood pressure per year differed; however, patients who started with dialysis were prescribed more antihypertensive drugs than those who received transplants (*P* = 0.03).

### Variables Associated With Initiation and Modality Choice of KRT

**Figure 2:**
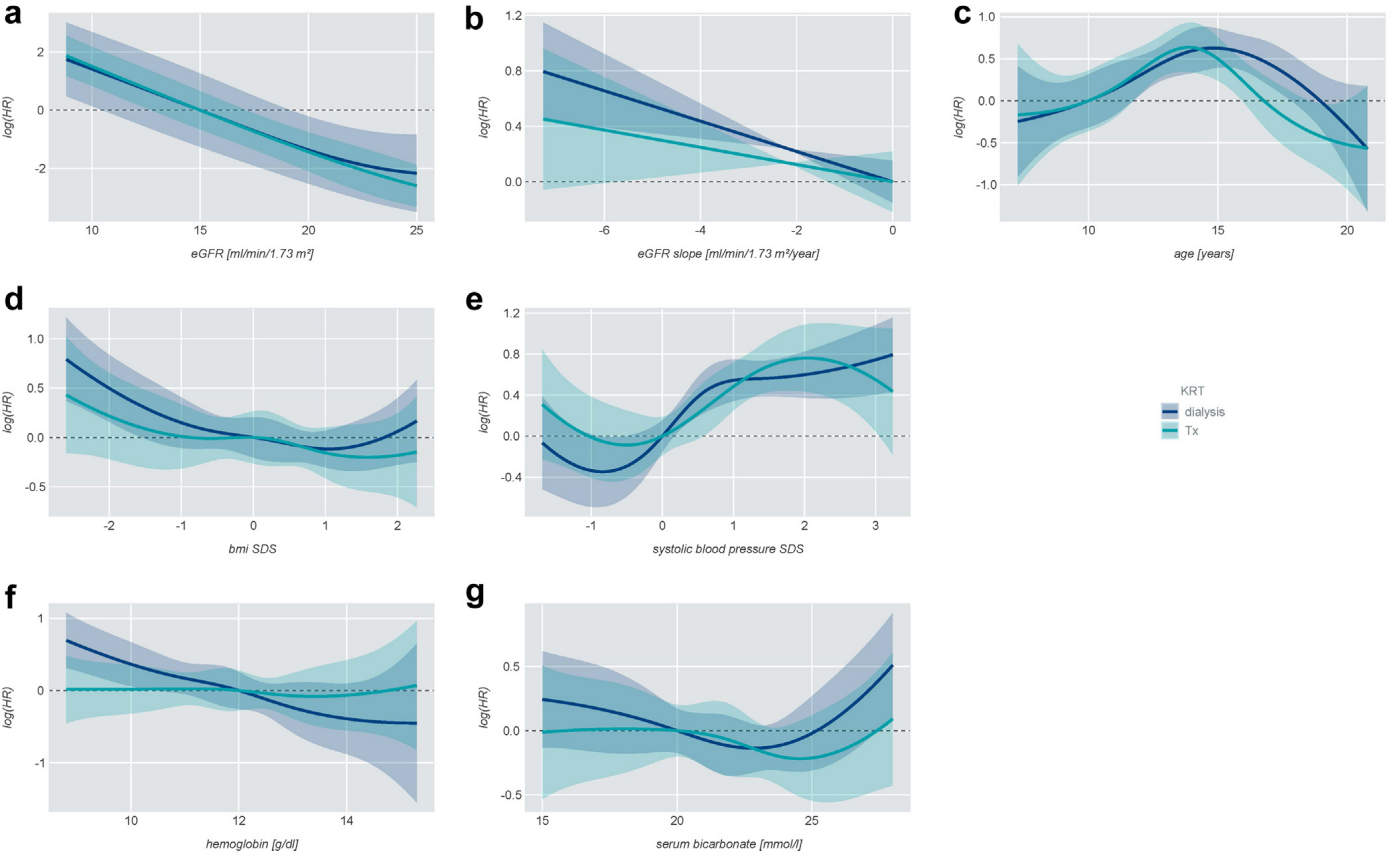
Nonlinear competing-risk for initiation of dialysis or preemptive transplantation according to (a) eGFR, (b) eGFR slope, (c) age, (d) BMI SDS, (e) systolic blood pressure SDS, (f) hemoglobin, (g) serum bicarbonate. y-axis shows the log (HR). The lines represent the mean effects (dark blue: dialysis; turquoise: preemptive transplantation) and shaded areas the respective 95% confidence intervals BMI, body mass index; eGFR, estimated glomerular filtration rate; HR, hazard ratio; SDS, SD score; Tx, transplantation.

eGFR and eGFR slope, primary renal diagnosis, comorbidities, age, systolic blood pressure SDS, BMI SDS, hemoglobin, and serum bicarbonate levels significantly contributed to the overall likelihood of starting KRT in the competing risk model (Figure 2 and Table 2).

**Table 2:** Factors associated with start of kidney replacement therapy

Variables	Any KRT		Dialysis		Preemptive Tx	
	HR (95% CI)	*P*	HR (95% CI)	*P*	HR (95% CI)	*P*
eGFR (ml/min per 1.73 m^2^)	0.76 (0.74–0.78)	<0.001	0.77 (0.74–0.8)	<0.001	0.74 (0.71–0.77)	<0.001
eGFR slope (ml/min per 1.73 m^2^ per yr)	0.90 (0.85–0.95)	<0.001	0.89 (0.83–0.96)	<0.001	0.93 (0.84–1.0)	0.14
Primary renal diagnosis (reference: CAKUT)						
Glomerulopathy	3.80 (2.51–5.75)	<0.001	5.39 (3.29–8.82)	<0.001	1.9 (0.83–4.38)	0.13
Tubulointerstitial	1.56 (1.14–2.14)	0.006	1.44 (0.94–2.20)	0.09	1.79 (1.11–2.88)	0.02
Post-AKI CKD	1.69 (0.94–3.03)	0.08	1.32 (0.61–2.86)	0.48	2.28 (0.95–5.49)	0.07
others/unknown	1.56 (0.85–2.86)	0.16	1.66 (0.76–3.6)	0.2	1.56 (0.59–4.14)	0.37
Comorbidities ≥ 1 vs. none	1.21 (0.913–1.6)	0.185	1.54 (1.05–2.24)	0.026	0.89 (0.587–1.35)	0.586
Age (reference: 6 to 9 yr)						
>9–12 yr	1.1 (0.66–1.83)	0.721	1.31 (0.67–2.57)	0.426	0.846 (0.38–1.89)	0.683
>12–14 yr	1.96 (1.19–3.22)	0.008	2.19 (1.13–4.23)	0.019	1.72 (0.806–3.68)	0.161
>14–16 yr	1.7 (1.03–2.81)	0.038	1.77 (0.90–3.49)	0.1	1.58 (0.74–3.36)	0.233
>16 yr	1.2 (0.728–1.97)	0.479	1.72 (0.90–3.29)	0.099	0.69 (0.313–1.51)	0.353
BMI SDS	0.77 (0.66–0.9)	0.001	0.73 (0.60–0.89)	0.002	0.82 (0.63–1.07)	0.151
Systolic blood pressure SDS (reference: −2 to 0)						
<−2	1.85 (0.769–4.44)	0.17	1.53 (0.45–5.19)	0.495	2.39 (0.67–8.5)	0.178
>0–1	1.62 (1.16–2.26)	0.005	1.83 (1.18–2.86)	0.007	1.34 (0.81–2.23)	0.258
>1	2.07 (1.49–2.87)	<0.001	2.15 (1.38–3.34)	<0.001	2.02 (1.23–3.32)	0.005
Hemoglobin (g/dl)	0.88 (0.81–0.96)	0.003	0.8 (0.72–0.9)	<0.001	1.00 (0.88–1.13)	0.954
Serum bicarbonate (mmol/l)	1.07 (0.998–1.14)	0.057	1.1 (1.01–1.21)	0.03	1.02 (0.91–1.14)	0.72

BMI, body mass index; CAKUT, congenital anomalies of the kidney and urinary tract; CI, confidence interval; eGFR, estimated glomerular filtration rate; HR, hazard ratio; KRT, kidney replacement therapy; post-AKI CKD, post-acute kidney injury chronic kidney disease; SDS, SD score; Tx kidney transplant.

Linear competing risk analysis. Only significant variables are shown. For some variables, correlation was linear only in definite ranges: eGFR ≤25 ml/min per 1.73 m^2^; BMI SDS ≤0; serum bicarbonate ≥22 mmol/l.

Patient age was associated with KRT initiation in an inverse U-shaped fashion (Figure 2c), dialysis likelihood being highest at about 16 years and transplant likelihood at about 14 years. Systolic blood pressure exhibited a sigmoidal association with KRT likelihood, with an inflection point around the 50th percentile (Figure 2d). In the piecewise linear approximation, a lower eGFR, a steeper eGFR slope, and having a glomerulopathy or tubulointerstitial disease increased the probability of starting any KRT (Figure 2 and Table 2). Patients aged 12 to 16 years were more likely to start KRT than those younger than 9 years. Higher systolic blood pressure SDS, lower BMI SDS, and lower hemoglobin levels increased the likelihood of starting KRT.

The likelihood of dialysis was increased by a lower eGFR, steeper eGFR slope, higher systolic blood pressure, and lower BMI SDS (Table 2). HRs were higher for patients with glomerulopathies in comparison to CAKUT and for patients with versus without comorbidities. The probability to start dialysis was also associated with lower hemoglobin and higher bicarbonate level (>22 mmol/l). Serum bicarbonate levels did not differ by center, primary renal diagnosis, and oral bicarbonate supplementation status (data not shown).

A lower eGFR and higher systolic blood pressure SDS increased the likelihood of preemptive Tx. Patients with tubulointerstitial diseases were more likely to receive a preemptive Tx than patients with CAKUT (Figure 2 and Table 2).

The decision to start KRT was subject to significant center effects, which accounted for 6.8% and 8.7% of the total explained variability for dialysis and preemptive Tx, respectively. Using a country effect instead of a center effect in the model, the effect contributes to a maximum of 2% increased variance explanation for dialysis and no effect is seen for preemptive Tx.

The significant random center effect in the model corresponded to HRs of 1.85 to 2.71 for the 15% of centers with the largest positive effect and 0.37 to 0.54 to the 15% centers with the largest negative effect (Table 3).

**Table 3:** Center effects on start of kidney replacement therapy

Center effect	Any KRT	Dialysis	Preemptive Tx
*P*-value	<0.001	<0.001	<0.001
Explained variation ΔR^2^_D_	6.4%	6.8%	8.7%
HR Top 15%	1.85	2.22	2.71
HR Min 15%	0.54	0.45	0.37

HR, hazard ratio; KRT, kidney replacement therapy; Tx, kidney transplant.

Confounding at center level was investigated using a frailty term. The impact of the center was determined by variation R^2^_D_. Second quantification of the center effect is shown as HR for the 15% of centers with the highest and lowest center effect respectively.

## Discussion

In this study, we explored factors associated with the decision to commence dialysis or perform preemptive Tx in a large prospective cohort study of children with CKD. The main determinant of the decision to start KRT was the level of eGFR. The median eGFR at start of dialysis or preemptive Tx was 11 and 10.7 ml/min per 1.73 m^2^, respectively. These values are substantially lower than the respective values reported in the North American CKD in Children study (17.8 and 19.1 ml/min per 1.73 m^2^),^21^ pointing to systematic regional variation in clinical practice. In both our study and the CKD in Children study, average eGFR at start of KRT was higher than reported in the population-based US Renal Data System, ERA-EDTA/ESPN, and Australia and New Zealand Dialysis and Transplant registries, where KRT was started at eGFRs of 7.8, 8.2, and 8 ml/min per 1.73 m^2^, respectively.^5^^,^^6^^,^^11^ The differences between the more recent study cohorts and the population averages could be related to the trend toward earlier initiation of KRT in the past 2 decades. Indeed, in the US Renal Data System database, eGFR at KRT start increased by 0.18 ml/min per 1.73 m^2^ per year.^5^ In that study, no patients with preemptive Tx were included.^5^ Larkins *et al.*^11^ also reported an increase of eGFR at KRT initiation in the Australia and New Zealand Dialysis and Transplant Registry, from 7 to 9 ml/min per 1.73 m^2^ between 1995 and 2018.^11^ The effect of era on median eGFR varied between KRT modalities, with a greater increase among those receiving a preemptive Tx (1.0 ml/min per 1.73 m^2^ per 5 years) or peritoneal dialysis (0.7 ml/min per 1.73 m^2^ per 5 years) compared with hemodialysis (0.1 ml/min per 1.73 m^2^ per 5 years).

In our cohort, eGFR equally determined the likelihood of starting dialysis or undergoing preemptive Tx. This finding is in keeping with data from the CKD in Children study, where median eGFR before initiation of dialysis and preemptive Tx were 17.8 and 19.1 ml/min per 1.73 m^2,^ respectively;^21^ whereas in the ERA-EDTA/ESPN registry, patients who started with preemptive Tx had a 3.4 ml/min per 1.73 m^2^ higher median eGFR compared with those starting on dialysis.^22^

The second most important determinant of KRT start was the rate of GFR loss. At a given eGFR, children with a more rapid eGFR decline were more likely to initiate KRT. This finding is in keeping with results of the CKD in Children study.^23^ Notably, the association was highly significant for initiation of dialysis but not for preemptive Tx, which was partially explained by the fact that glomerulopathies, the disorders associated with most rapid progression, were underrepresented in the latter group. In addition, the greater flexibility of timing when a transplant donor is available facilitates the initiation of KRT primarily according to absolute eGFR level, with less dependence on the rate of eGFR deterioration.

When adjusting for both absolute eGFR and the rate of eGFR loss in the model, several additional factors affecting the likelihood of KRT initiation became apparent. The most evident factor influencing the start of KRT was the primary renal diagnosis. Patients with underlying glomerulopathies were more than 5 times more likely to start dialysis than patients with CAKUT, whereas their likelihood to undergo preemptive Tx was not increased. This may be due to the risk of posttransplant disease recurrence and the common policy to postpone Tx in this group of patients.

The presence of several comorbidities increased the likelihood to start dialysis but not preemptive Tx, possibly reflecting the perceived greater risk of clinical decompensation in these patients.

Standardized BMI was an inverse risk factor for the start of dialysis. The likelihood of initiating dialysis independently increased by 27% per 1 SD decrease in BMI. Although there were few children with overt malnutrition, it appears that the decision to initiate dialysis may have been influenced by poor weight gain. In notable contrast, growth failure had no significant impact on the likelihood of KRT, which may reflect the understanding that growth failure typically does not improve by starting dialysis but is more efficiently treated with recombinant growth hormone, which is more efficacious in predialysis CKD than on dialysis.^24^

Higher systolic blood pressure was a predictive factor for both dialysis and preemptive Tx. The association was already seen for blood pressure levels in the upper normal range. Patients with systolic blood pressure above the 85th percentile were twice as likely to initiate KRT by either dialysis or preemptive Tx than children with low-normal blood pressure, independently of eGFR, the underlying kidney disorder, and the current CKD progression rate. Blood pressure increases in the late pre-KRT period usually indicate increasing salt and fluid retention. Our study suggests that clinicians pay close attention to blood pressure increases and take even moderate changes into account when deciding to initiate KRT in the participating centers in Europe and Türkiye.

The likelihood of initiating dialysis was also affected by the degree of anemia. Although mean hemoglobin levels at start of KRT were within the target range, there was substantial individual variation, and the chance to commence dialysis increased by 20% with each 1 g/dl lower hemoglobin. Treatment refractory anemia in the late pre-KRT phase may reflect erythropoiesis-stimulating agent resistance and/or dilution secondary to fluid overload. Either interpretation may contribute to the clinical decision to launch dialysis.

An unexpected finding was the fact that serum bicarbonate levels above 22 mmol/l, rather than acidosis, were positively associated with initiation of dialysis. This association, which was not explained by underlying disease distribution, the use of bicarbonate medication or center effects, was of borderline statistical significance and questionable clinical relevance.

Next to the conditions identified as risk factors for starting KRT, it is worth mentioning which factors were not associated with the decision to commence dialysis or perform preemptive Tx. Sex and ethnicity, factors associated with transplant access in previous pediatric studies,^25^, ^26^, ^27^ did not contribute to the variance of the model.

Likewise, CKD mineral bone disorder activity as indicated by parathyroid hormone, serum calcium, and serum phosphorus levels did not associate with the likelihood of KRT initiation. Although clinicians are certainly aware of the relevance of childhood CKD mineral bone disorder for long-term bone and cardiovascular health, hyperphosphatemia, and hyperparathyroidism, this might not be a major factor influencing the decision to start KRT.^28^^,^^29^ Likewise, serum urea and potassium levels did not appear to drive KRT decision-making. The results of this study should not be influenced by the prospect of a living donation, because only 3% of patients received a living donation within 3 months after starting dialysis.

Finally, we identified significant center effects, which grossly amounted to a doubling of KRT likelihood in the 15% most KRT-affine and a halved likelihood in the 15% most restrained centers, independent of eGFR, disease progression rate, and all other clinical and biochemical factors listed above. The variation of center practice equally related to dialysis and preemptive Tx and was attributable to the individual centers rather than the countries where the centers were located.

A major strength of this study is the size of the cohort enrolled and followed prospectively, with more than 50% reaching the KRT endpoint. In addition, our study is the first to compare predictive factors for dialysis and preemptive Tx. Limitations of the study are given by the study design, which did not include an additional visit at the time of starting KRT. This limited the precision of the patient characterization at KRT start according to time-dependent anthropometric and biochemical parameters. Another limitation is given by the fact that access to preemptive deceased donor Tx varies widely between countries and continents.^30^^,^^31^ Therefore, not all results might be globally applicable. In addition, creatinine-based eGFR equations such as the Schwartz bedside formula used here were developed in participants with eGFR > 15 ml/min per 1.73 m^2^ and may be confounded by low muscle mass. This may influence the results with respect to kidney function.

In conclusion, we identified in a comprehensive statistical approach, factors associated with the decision to initiate dialysis or perform preemptive Tx in children and adolescents with kidney failure. Although eGFR was the main determinant of commencing any KRT, the rate of eGFR loss, adolescent age, the presence of a glomerulopathy, wasting, as well as poor blood pressure and anemia control increased the likelihood of initiating dialysis. On top of these measurable factors, substantial center variation was observed, pointing to major differences in physicians’ general attitudes toward starting KRT in pediatric patients.

## Disclosure

FS and AM - support from the ERA-EDTA Research Program, the KFH Foundation for Preventive Medicine and the German Federal Ministry of Education and Research (01EO0802); European Reference Network for Rare Kidney Diseases (10.13039/501100021081ERKNet); Roche Organ Transplant Research Foundation; the other authors have no disclosures.

## Appendix

### List of 4C Study Group

Austria: G. Cortina, Children’s Hospital, Innsbruck; K. Arbeiter, University Children’s Hospital, Vienna. Czech Republic: J. Dusek, University Hospital Motol, Prague. France: J. Harambat, Hôpital des Enfants, Bordeaux; B. Ranchin, Hôpital Femme Mère Enfant et Université de Lyon; M. Fischbach, A.Zalosczyk, Hôpital de Hautepierre, Strasbourg. Germany: U. Querfeld, Charité Children’s Hospital, Berlin; S.Habbig, University Children’s Hospital, Cologne; M. Galiano, University Children’s Hospital, Erlangen; R. Büscher, University Children’s Hospital, Essen; C. Gimpel, Center for Pediatrics and Adolescent Medicine, Freiburg; M. Kemper, UKE University Children’s Hospital, Hamburg; A. Melk, D. Thurn, Hannover Medical School, Hannover; F. Schaefer, A. Doyon, E. Wühl, Center for Pediatrics and Adolescent Medicine, Heidelberg; M. Pohl, Center for Pediatrics and Adolescent Medicine, Jena; S. Wygoda, City Hospital St. Georg, Leipzig; N. Jeck, KfH Kidney Center for Children, Marburg; B. Kranz, University Children’s Hospital, Münster; M. Wigger, Children’s Hospital, Rostock. Italy: G. Montini, S. Orsola-Malpighi Hospital, Bologna; F. Lugani, Istituto Giannina Gaslini, Genova; S. Testa, Fondazione Ospedale Maggiore Policlinico, Milano; E. Vidal, Pediatric Nephrology, Dialysis & Transplant Unit, Padova; C. Matteucci, S. Picca, Ospedale Bambino Gesù, Rome. Lithuania: A. Jankauskiene, K. Azukaitis, University Children’s Hospital, Vilnius. Poland: A. Zurowska, Pediatric and Adolescent Nephrology, Gdansk; D. Drodz, University Children’s Hospital, Krakow; M. Tkaczyk, Polish Mothers Memorial Hospital Research Institute, Lodz; T. Urasinski, Clinic of Pediatrics, Szczecin; M. Litwin, A.Niemirska, Children’s Memorial Health Institute, Warsaw; M. Szczepanska, Zabrze. Portugal: A. Texeira, Hospital Sao Joao, Porto; Serbia: A. Peco-Antic, University Children’s Hospital, Belgrade. Switzerland: B.Bucher, Inselspital, Bern; G. Laube, University Children’s Hospital, Zurich. Türkiye: A. Anarat, A.K. Bayazit, Cukurova University, Adana; F. Yalcinkaya, University Faculty of Medicine, Ankara; E. Basin, Baskent University Faculty of Medicine, Ankara; N. Cakar, Diskapi Children’s Hospital, Ankara; O. Soylemezoglu, Gazi University Hospital, Ankara; A. Duzova, Y. Bilginer, Hacettepe Medical Faculty, Ankara; H. Erdogan, Dortcelik Children’s Hospital, Bursa; O. Donmez, Uludag University, Bursa; A. Balat, University of Gaziantep; A. Kiyak, Bakirkoy Children’s Hospital, Istanbul; S. Caliskan, N. Canpolat, Istanbul University Cerrahpasa Faculty of Medicine, Istanbul; C. Candan, Goztepe Educational and Research Hospital, Istanbul; M. Civilibal, Haseki Educational and Research Hospital, Istanbul; S. Emre, Istanbul Medical Faculty, Istanbul, H. Alpay, Marmara University Medical Faculty, Istanbul; G. Ozcelik, Sisli Educational and Research Hospital, Istanbul; S. Mir, B. Sözeri, Ege University Medical Faculty; Izmir; O. Yavascan, Tepecik Training and Research Hospital, Izmir; Y. Tabel, Inonu University, Malatya; P. Ertan, Celal Bayar University, Manisa; E. Yilmaz, Children’s Hospital, Sanliurfa. United Kingdom: R. Shroff, Great Ormond Street Hospital, London.

## References

[1] Preka E., Rees L. (2020). Should we abandon GFR in the decision to initiate chronic dialysis?. Pediatr Nephrol.

[2] Mitsnefes M.M. (2012). Cardiovascular disease in children with chronic kidney disease. J Am Soc Nephrol.

[3] Schmidt B.M.W., Sugianto R.I., Thurn D. (2018). Early effects of renal replacement therapy on cardiovascular comorbidity in children with end-stage kidney disease: findings from the 4C-T study. Transplantation.

[4] Okuda Y., Soohoo M., Tang Y. (2019). Estimated GFR at dialysis initiation and mortality in children and adolescents. Am J Kidney Dis.

[5] Winnicki E., Johansen K.L., Cabana M.D. (2019). Higher eGFR at dialysis initiation is not associated with a survival benefit in children. J Am Soc Nephrol.

[6] Preka E., Bonthuis M., Harambat J. (2019). Association between timing of dialysis initiation and clinical outcomes in the paediatric population: an ESPN/ERA-EDTA registry study. Nephrol Dial Transplant.

[7] Cooper B.A., Branley P., Bulfone L. (2010). A randomized, controlled trial of early versus late initiation of dialysis. N Engl J Med.

[8] Crews D.C., Scialla J.J., Boulware L.E. (2014). Comparative effectiveness of early versus conventional timing of dialysis initiation in advanced CKD. Am J Kidney Dis.

[9] Clark W.F., Na Y., Rosansky S.J. (2011). Association between estimated glomerular filtration rate at initiation of dialysis and mortality. CMAJ.

[10] Liu Y., Wang L., Han X. (2020). The profile of timing dialysis initiation in patients with end-stage renal disease in china: a cohort study. Kidney Blood Press Res.

[11] Larkins N.G., Lim W., Goh C. (2023). Timing of kidney replacement therapy among children and young adults. Clin J Am Soc Nephrol.

[12] Daugirdas J.T., Depner T.A., Inrig J. (2015). KDOQI clinical practice guideline for hemodialysis adequacy: 2015 update. Am J Kidney Dis.

[13] Querfeld U., Anarat A., Bayazit A.K. (2010). The cardiovascular comorbidity in Children with Chronic Kidney Disease (4C) study: objectives, design, and methodology. Clin J Am Soc Nephrol.

[14] Schwartz G.J., Muñoz A., Schneider M.F. (2009). New equations to estimate GFR in children with CKD. JASN.

[15] Therneau T.M., Grambsch P.M. (2023). Modeling Survival Data: Extending the Cox Model.

[16] Bates D., Mächler M., Bolker B., Walker S. (2015). Fitting linear mixed-effects models using lme4. J Stat Softw.

[17] Royston P., Sauerbrei W. (2004). A new measure of prognostic separation in survival data. Stat Med.

[18] Buuren S van, Groothuis-Oudshoorn K. (2011). mice: multivariate Imputation by Chained Equations in R. J Stat Softw.

[19] R: the R Project for Statistical Computing. https://www.r-project.org/.

[20] Therneau T.M. Survival Analysis [R package survival version 3.7-0]. https://cran.r-project.org/web/packages/survival/index.html.

[21] Atkinson M.A., Roem J.L., Gajjar A., Warady B.A., Furth S.L., Muñoz A. (2020). Mode of initial renal replacement therapy and transplant outcomes in the chronic kidney disease in children (CKiD) study. Pediatr Nephrol.

[22] van Stralen K.J., Tizard E.J., Jager K.J. (2010). Determinants of eGFR at start of renal replacement therapy in paediatric patients. Nephrol Dial Transplant.

[23] Zhong Y., Muñoz A., Schwartz G.J., Warady B.A., Furth S.L., Abraham A.G. (2014). Nonlinear trajectory of GFR in children before RRT. J Am Soc Nephrol.

[24] Haffner D., Rees L., Schaefer F., Greenbaum L.A. (2023). Pediatric Kidney Disease.

[25] Tjaden L.A., Noordzij M., van Stralen K.J. (2016). Racial disparities in access to and outcomes of kidney transplantation in children, adolescents, and young adults: results from the ESPN/ERA-EDTA (European society of Pediatric Nephrology/European Renal Association-European Dialysis and transplant association) registry. Am J Kidney Dis.

[26] Patzer R.E., Sayed B.A., Kutner N., McClellan W.M., Amaral S. (2013). Racial and ethnic differences in pediatric access to preemptive kidney transplantation in the United States. Am J Transplant.

[27] Hogan J., Couchoud C., Bonthuis M. (2016). Gender disparities in access to pediatric renal transplantation in Europe: data from the ESPN/ERA-EDTA registry. Am J Transplant.

[28] Lalayiannis A.D., Soeiro E.M.D., Moysés R.M.A., Shroff R. (2023). Chronic kidney disease mineral bone disorder in childhood and young adulthood: a “growing” understanding. Pediatr Nephrol.

[29] Bacchetta J., Schmitt C.P., Bakkaloglu S.A. (2023). Diagnosis and management of mineral and bone disorders in infants with CKD: clinical practice points from the ESPN CKD-MBD and Dialysis working groups and the Pediatric Renal Nutrition Taskforce. Pediatr Nephrol.

[30] Harambat J., van Stralen K.J., Schaefer F. (2013). Disparities in policies, practices and rates of pediatric kidney transplantation in Europe. Am J Transplant.

[31] Bonthuis M., Cuperus L., Chesnaye N.C. (2020). Results in the ESPN/ERA-EDTA registry suggest disparities in access to kidney transplantation but little variation in graft survival of children across Europe. Kidney Int.

